# Development of Mother’s Lifestyle Scale during Pregnancy with an Approach to Social Determinants of Health

**DOI:** 10.5539/gjhs.v5n3p208

**Published:** 2013-03-31

**Authors:** Zohreh Mahmoodi, Masoud Karimlou, Homeira Sajjadi, Masoumeh Dejman, Meroe Vameghi

**Affiliations:** 1 Social Determinant of Health Research Center, University of Social Welfare and Rehabilitation Sciences, Tehran, Iran

**Keywords:** lifestyle, development of measurement instruments, psychometrics, social determinants of health

## Abstract

**Background and Objective:**

The present study was conducted to design and measure psychometrics of mothers’ lifestyle scale during pregnancy with regards to Iranians’ cultural norms and an approach to social determinants.

**Method:**

this study, started by reviewing previous studies and exploring similar questionnaires that examine different domains of lifestyle (nutrition, exercising, self-care, smoking, using alcohol and illegal drugs, social relations, and stress control), then besides translating questions of the questionnaires, content of some questions was modified and proper statements with regards to social determinant of health and Iranian cultural, was used. Secondly, the validity of the designed instruments was determined using face, content, criterion, and construct validity. Thirdly, the reliability of the measurement instruments was examined using Cronbach’s alpha. Participants were Healthy Iranian pregnant women (37-42 week) who refer to selected hospital for delivery.

**Results:**

In the first step of the study, of the 222 questions obtained from a review of the related instruments, certain questions were omitted due to their irrelevance to social determinants of health and finally 160 questions were selected in 10 sections. After determining the face and content validity qualitatively and quantitatively and exploratory factor analysis, the number of questionnaire items was reduced to 132. Calculation of Cronbach’s alpha confirmed the high internal consistency (0.76) of the questionnaire.

**Conclusion:**

This measurement instrument was designed in the context of the Iranian culture and sounded suitable for studying the pregnant women’s lifestyle due to its appropriate validity and reliability, simplicity, and functionality in different situations.

## 1. Introduction

The health of women and pregnant mothers as one of the most vulnerable groups is of special importance in health care system (Yazdi & Mahjoub, 2011). As today, one of the key indices of health in every country is pregnant women’s mortality which directly or indirectly results from pregnancy and its complications. Pregnancy and delivery are not diseases; however, they have potential risks which can be reduced through interventions like health care (WHO, 2009). Therefore, identifying the factors that play a role in causing or preventing problems during this period is very critical (Adlshoar et al., 2006). Many studies have stated a correlation between dimensions of mothers’ lifestyle (nutrition, smoking, and exercising) during pregnancy and unfavorable outcomes like premature delivery (Mosayebi et al., 2004; Alexander et al., 2007; Talebian & Afrouz, 2011). In the initial discussions, lifestyle was primarily focused on nutrition, physical activity, smoking, and alcohol use. However, today, people’s perception of lifestyle and its relation to health has been subjected to major changes, and studies and experiences in the area of health promotion have changed people’s way of thinking about lifestyle and their attempts to promote health. A revised definition of lifestyle must account the effect of social conditions and processes such as social-economical status and social relations on the lifestyle besides factors affecting nutrition, physical activity, and alcohol use (Rafieifar & Damari, 2005). Accordingly, in his study, MacDonald (2010) introduces the dimensions of lifestyle as nutrition, exercising, self-care, smoking, consumption of alcohol and illegal drugs, social relations, and control of stress (McDonald & Thompson, 2005).

In order to study any subject, researchers need instruments related to that subject to collect required information with maximum accuracy and minimum errors (Eslami et al., 2011). However, the analysis of previous studies showed that 1) no study has been conducted on the basis of WHO’s model with an approach to social determinants of health in Iran 2) so far, the instruments applied to examine lifestyle were not specific to pregnancy or were used for special diseases like cardiovascular diseases and diabetes, or, 3) they were not conducted with an approach to the social determinants of health. In fact, majority of those studies had biological attitudes and were looking for mechanisms associated with a specific area and its relevant factors (Bae, 2011; Monzillo et al., 2012), 4) the majority of instruments were conducted on the basis of one of lifestyle like physical activity/stress in pregnancy. In our study we collected most aspects of lifestyle (Occupational lifestyle, nutrition, stress control, unsafe behavior (smoking and drug use), self-care, social relations) with the social determinants of health approach.

Whereas, today, regarding the role of social determinants of health which are seeking the first cause of diseases, designing a questionnaire with the reliability and validity consistent with Iranian religion and culture to study pregnant women’s lifestyle has become important more than ever. Therefore, purpose of this study was to designing an instrument with this approach and perhaps takes a small step toward mothers’ health.

## 2. Methods

For designing a measurement instrument, statement can be designed from literature review, qualitative research like grounded theory, selecting items from available instruments or mixed of methods (Frank-Stromborg, 2004). In study 1, designing and selecting appropriate items for the measurement were carried out after reviewing previous studies and exploring similar questionnaires that could facilitate determining the desired instrument. Considering that the research topic was new and no similar study was found elsewhere, attempts were focused on questionnaires which have taken account of the above-mentioned dimensions of lifestyle. To be loyal to the content of the questionnaires, they were first translated into Persian and then back translated into English. Based on the experts’ opinions, questions appropriate for measuring pregnant women’s lifestyle with an approach to social determinants of health. Finally, the initial questionnaire was designed and then, the items were assessed by the relevant experts and specialists in order to determine whether the questions measured exactly what they must measure. It is noteworthy that besides translating questions of the questionnaires and modifying the content of some questions and using proper statements with regards to cultural differences between Iran and communities of the applied questionnaires. The questions were localized within 7 sessions of group discussions with experts and them was changed or cut if wasn’t the social determinants of health and Iranian religion and culture.

### 2.1 Study 1-Determining Measurement Instruments

The questionnaires applied for designing the initial questionnaire were as follows: 1) Beck’s Physical Activity Questionnaire (Baecke, Burema, & Frijters. 1982). 2) PRAM^1^, (Brown et al., 2011), 3) lifestyle questionnaire (www.wellnesswizards.com), 4) lifestyle questionnaire (Kamali Fard et al., 2010), 5) access-health lifestyle survey (section were obtained from the Health Promoting Lifestyle Profile II by permission and access in www.access-health.org), and 6) Bell Adjustment Inventory (rambell, 1961). Based on the above questionnaires, the initial questionnaire with 160 items was prepared considering the attitude of social determinants of health and Iranian religion/culture by the researchers (in the field of statistics, social medicine, psychiatry, nursing, and social determinants of health) within 7 sessions of group discussions. In this step one item related to social determinant of health and Iranian religion and culture was presented to panel members.

The designed questionnaire included 10 sections: general specifications, a history of pregnancy, and laboratory results recorded in mothers’ file (37 items) and 7 domains were about mothers’ lifestyle which included:

Occupational lifestyle, nutrition, stress control, unsafe behavior (smoking and drug use), self-care, social relations measured with the Likert scale from 1 to 5 (the most favorable to the most unfavorable), and physical activity (including physical activity at home, at free times, and exercising) measured with MET^2^ scale (the method to measure physical activity) (Jette et al., 1990; Ainsworth et al., 2011).

#### 2.1.1 Psychometric Analysis of the Instruments

In the first study, Validity of the designed instruments was determined using face validity, content validity, criterion validity, and the second study has done construct validity.

#### 2.1.2 Face Validity

Validity has both qualitative and quantitative aspects. The qualitative aspects are conceptual. The quantitative aspects are numerical (Wright & Stone 1999). Thus for all domains of questionnaire both aspects were done. In this study, we used each participant for both qualitative and quantitative aspects. It means every participant answered qualitative and quantitative aspects, respectively.

At first, in order to determine the face validity qualitatively, 10 pregnant women (Wilson, 1989) admitted to one of the hospitals in Tehran, Iran (Shahid Akbarabadi hospital) were interviewed face to face, and difficulty level of items (difficulty in understanding statements and words) was examined (flowchart 1). Once the items were modified according to the pregnant women’s opinions. quantitative method of impact score was used to reduce items, delete inappropriate statements and determine the importance of each statement for this we want of them (previous pregnant women) to chose one code of different 5-point likert scale (i.e., 5=strongly important,1= strongly not important). In this respect, according the formula of Impact Score (Frequency (%)ÏImportance), was calculated, the statements’ impact scores which were equal to or greater than 1.5 were maintained for the next analysis (Lacasse et al., 2002).

**Flowchart:**
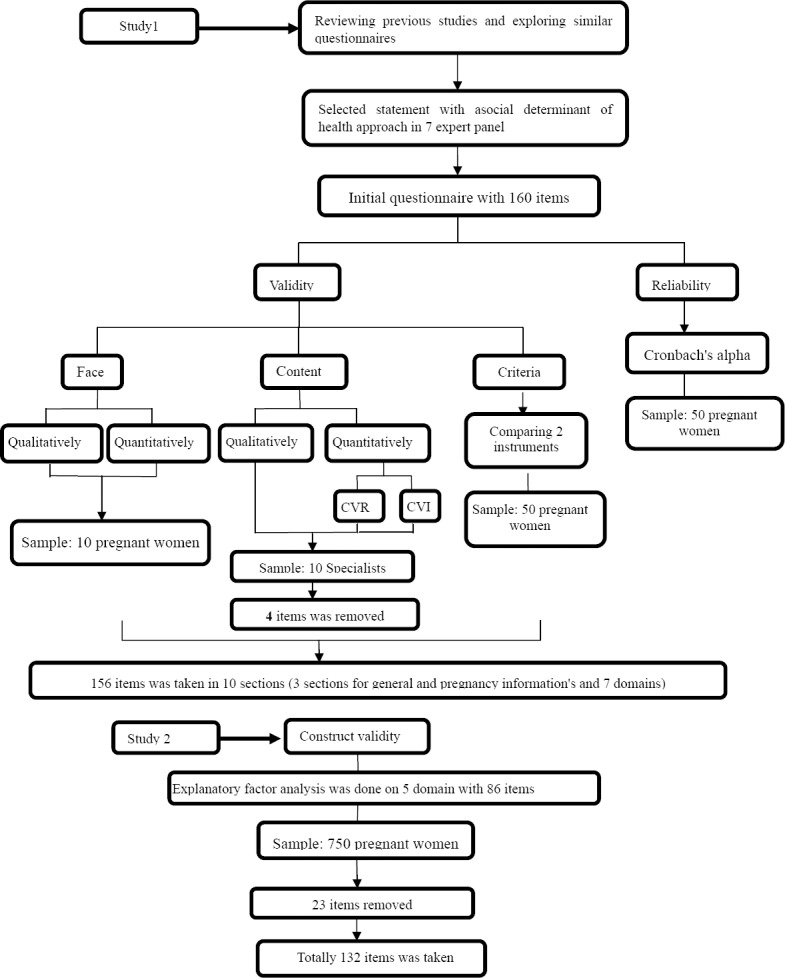
Step of development and psychometrics of mother’s lifestyle scale during pregnancy with an approach to social determinants of health

#### 2.1.3 Content Validity

Content validity was also determined qualitatively and quantitatively. In this study, content validity was determined based on the judgments of specialists in the fields of measurement instrument designing, psychiatry, social medicine, obstetrics, nutrition, and Ph.D. in nursing. For the qualitative validation of content, the researchers asked 10 specialists in the above-mentioned fields (Wilson, 1989) to provide the necessary feedback after qualitative analysis of the questionnaire in terms of grammar, wording, necessity, significance, item allocation, and scaling. For the quantitative validation of content, 2 indices of content validity ratio (CVR) and content validity index (CVI) were used. To determine CVI, 10 previous specialists were asked to analyze each item on the basis of a 3-point Likert scale (It is necessary, It is useful but not necessary, It is not necessary). If the value obtained from Lawshe table for determining the minimum value of the index was greater than 0.62 (based on the assessment of the 10 specialist), the item with the statistical significance level of P<0.05 was taken as important and necessary for the measurement instrument (Lawshe, 1975(. Then, CVI was determined based on Waltz and Basel content validity index. In this respect, 10 specialists in the relevant field were requested to determine the relevance, simplicity, and clarity of each item of the questionnaire. These three criteria were assessed separately for each item in a 4-point Likert scale. If the CVI value of an item was greater than 0.79, the item would be taken as appropriate, if the CVI value of an item was 0.70-0.79, the item would be questionable, and if the CVI value of an item was lower than 0.70, the item would be unacceptable and would be deleted (Polit et al., 2007) (flowchart 1). Then, mean CVI (S-CVI/Ave^3^) of the questionnaire was calculated based on mean CVI of all statements of the questionnaire. Polit and Beck have suggested the score equal to or greater than 90% for an acceptable S-CVI/Ave (Polit et al., 2006).

#### 2.1.4 Criterion Validity

To determine the criterion validity, the mentioned measurement instruments were compared to the instruments which were valid and reliable for examining the lifestyle and premature delivery in Iran (Kamali Fard et al., 2010). The instrument used in Iran consists of 42 questions in 8 sections: demographics and pregnancy-related information, and 6 domains on mothers’ lifestyle including exercising, nutrition, alcohol and drug use, social relations during pregnancy, stress control, and self-care during pregnancy. After comparison of the two sets of instrument in 50 pregnant women, the correlation coefficient of values obtained from the two sets of instrument was calculated as the predictive validity index of the test (flowchart 1). In this respect up to this stage 156 statement was taken for next analysis.

### 2.2 Study 2- Construct Validity

Given that the measurement instrument of this study was designed for the first time, the exploratory factor analysis was employed in order to determine the construct validity and explain the correlation patterns between the statements of each area (Vakili et al., 2012). Various studies consider 5-10 samples per statement and some of them even mention 3 samples per statement enough for performing the exploratory factor analysis (Munro, 2005). from 10 sections: general specifications, a history of pregnancy, and laboratory results recorded in mothers’ file (37 items), unsafe behaviors domain (11 items) that were in the form of a checklist, and physical activity domain (11 items) which was numerical and based on MET scale were removed. The exploratory factor analysis is often used as a data reduction technique with ordered categorical indicators and dichotomous items scored 0-1. It would not be appropriate with non-orderable categorical indicators with more than 2 categories and also it is good for interval variable but physical activity domains measured by MET that is nominal and rational indicator so we couldn’t do factor analysis for this. Unsafe behaviors domain (smoking and drug use), are checklist. Answering to these questions was limited to yes/no. therefore, because of clarity of questions; we didn’t need to factor analysis. Demographic and pregnancy history (37 item), which are recorded in mothers file, usually use for all patients in hospital; thus it is clear and doesn’t need factor analysis. This part considered as separate part from questionnaire (Chahouki, 2009; Forero, Maydeu-Olivares, & Gallardo-Pujol, 2009).

Therefore, the number of items used for the exploratory factor analysis, was 86 in the other 5 domains. The number of samples (pregnant women) was determined 8 times as the number of items and therefore 750 participants were selected considering 10% downfall. Samples were randomly selected from Tehran’s selected hospitals. To do so, at first, Tehran was divided into 5 regions of north, south, east, west, and center. In each region, 1 hospital (educational or affiliated to Social Security Organization) was selected randomly. Then, based on the pregnancy statistics of every hospital, the required samples were selected randomly. Participants were:

1-Iranian pregnant women aged 15-45 years who were 37-42 gestational age and referred for delivery to selected hospital
2-Without any kind of medical limitation (such as preeclampsia, endocrinal and psychological diseases or using drug effects on birth weight).
3-They desired and stabled to participate and answer the questionnaire of the study. The researcher or trained questioners filled the questionnaire through interviewing the pregnant women and reviewing their medical records.

#### 2.2.1 Reliability of the Measurement Instrument

Reliability of the questionnaire was determined using internal consistency. Cronbach’s alpha is the most common method for examining the internal consistency of instruments (Polit et al., 2006). For In this part, 50 samples, randomly selected to examine the internal consistency of the instrument. After collecting the 50 questionnaires distributed among the research units, internal consistency of the instrument (Cronbach’s alpha) was determined equal to or greater than 0.7. Finally, the questionnaire was confirmed to be widely used in the study.

In order to respect the ethical considerations, the study was conducted upon receiving the consent of the presidents of University of Tehran, Shahid Beheshti University, and General department of Social Security for their affiliated hospitals. Moreover, prior to the study, the pregnant women signed an informed consent form after they were aware of the objectives of the study and assured that their information would remain confidential and they could withdraw from the study whenever they liked.

The study was approved by Welfare and Rehabilitation Sciences University and research center for social determinant of health Ethics Committee.

## 3. Results

In this study, 750 pregnant women with mean age of 27.51±5.2 years were examined. Table 1 shows specifications of the participants.

**Table 1:** Frequency distribution of age groups, education, age and occupation of mothers in 5 regions of Tehran

Mother's specifications	Frequency (%)	
**Age**	<20	45(6%)
	20-35	657(87/6%)
	>36	48(6/4%)
**Level of education**	0	13(1/7%)
	1-6	110(14/7%)
	7-12	529(70/5%)
	13-16	83(11/1%)
	>17	15(2%)
**Occupational status**	Employed	91(12/1%)
	Housewife	659(87/9)
**Numbers of pregnancy**	1	352(46/9%)
	2-3	349(46/5%)
	>4	49(6/5%)

**Face validation:** The results of the qualitative face validation led to changes in the content of some items. However, as the impact score of any statement was not less than 1.5, no statement was deleted.

**Content validation:** In qualitative content validation, some modifications were made based on the experts’ opinions. However, in quantitative content validation, 4 statements (in the areas of occupation and physical activity) were deleted due to their CVR value less than 0.62. According to the results of CVI, mean relevance, mean clarity, mean simplicity, and S-CVI/Ave comprised 96.91±0.67, 96.73±0.70, 97.64±0.66, and 97.09±0.63, respectively. None of the statements was deleted in this step, so 156 statements were qualified for the next step.

**Criterion validation:** After sampling and determining the correlation coefficient of the values of the two sets of instruments, the correlation between the two sets of instruments was confirmed (p=0.026, r=0.4).

**Construct validation:** The exploratory factor analysis was performed on 86 statements using principal components. The KMO index was 0.92 in the exploratory factor analysis (Bartlett X2=14304, df=1326, p=0.000). Using Kaiser Criterion and un rotated factors (Eigen value>1), 5 factors were extracted. The method justified the 44% of the scale variance. Once the factors and their items were extracted, the conceptual consistency of the items allocated to the factors was examined. In this respect, 23 items in the areas of nutrition (11), stress (7), self-care (1), and social relations (4) were deleted due to their inconsistency with each other or the factor loading less than 0.3. The structure of factors and their loadings, Eigen value, and percentage of variance that were explained by each factor are shown in Table 2. Finally, the number of items became 132 by adding questions related to demographic specifications, physical activity, and unsafe behaviors.

**Table 2:** Results of the exploratory factor analysis, the extracted factors, Eigen value, the predicted percentage of variance, and Cronbach's alpha

Statements of lifestyle measurement instruments	Factor 1 (occupation)	Factor 2 (self-care)	Factor 3 (nutrition)	Factor 4 (stress)	Factor 5 (social Rlations)
Radioactive materials	.975				
Moisture	.974				
Noise	.975				
Detergents	.974				
Lifting heavy objects	.975				
Computer	.975				
Standing	.980				
Sitting	.980				
Walking	.980				
Were you satisfied with your workplace?	.980				
How tired were you after work?	.980				
To what extent, did you feel the authorities at your workplace realize your condition?	.980				
What do you think about the amount of your activities at work during pregnancy?	.980				
What was your work shift?	.980				
Stress at workplace	.849				
Did you have the three main meals (breakfast, lunch, and dinner) daily?		.581			
Did you eat enough food?		.469			
Did you go to the hospital for prenatal care at recommended times?		.320			
Did you do the routine prenatal tests?		.440			
Did you visit a doctor when you became sick (physically or mentally)?		.564			
Did you visit a dentist in case of feeling any discomfort in your mouth or teeth during pregnancy?		.388			
Did you use any chemical medicines without doctor's prescription?		.423			

Did you use any herbal medicines during pregnancy (essences and brewed herbs)?		.463			
Did you avoid contacting hazardous chemicals (household cleaners, pesticides, dyes)?		.320			
Did you take vitamin and mineral supplements like folic acid, iron, and multivitamins according to doctor's prescription?		.529			
Stress in the family environment		.442			
Moving to a new house or place?		.313			
To what extent did you use mobile phone for calling others during pregnancy?		.334			
Do you think you had a healthy diet during your pregnancy?			.507		
Did you consume snacks daily?			.587		
Milk (4 glasses)			.569		
Dairy (4 glasses)			.632		
Meat (3-4 units)			.525		
Cereals (one cup)			.376		
Consumption of chicken (3-4 units)			.368		
Weekly consumption of fish			.333		
Did you reduce consumption of fats, animal oils, and margarine (up to 20-30 g of butter, 2-3 tablespoons vegetable oil or margarine, removing meat's fat, consumption of low-fat milk, and reducing the use of fatty sauces) in the last trimester of pregnancy?			.331		
Nuts (pistachios, walnuts, hazelnuts, etc.)			.367		
How stressed were you during pregnancy?				.534	
How stressed were you while driving automobiles during pregnancy?				.314	
To what extent, could you express your emotions?				.511	
Any diseases or severe injuries?				.486	
Did any diseases or severe injuries happen to family members?				.371	
Death of close relatives				.364	
Separation from spouse				.356	
Emotionally abused or humiliated by the spouse or others				.333	
Physical damages by the spouse				.392	
Becoming homeless				.687	
Loss of Job				.707	
Loss of husband's Job				.839	
Husband addicted to drugs				.844	
Physical damages by others				.379	
Decision-making for household affairs (shopping, replacing equipment, etc.)					.378
Decision-making for personal affairs					.446
How did you feel happy and satisfied in your family environment?					.579
To what extent did you feel the sympathy of person(s) living with you?					.588
To what extent did the personal habits of person(s) living with you make you irritated or sad?					.580
How easy was it for you to cope with person(s) living with you?					.541
To what extent did you feel shy when you entered or exited from a strange place or group of people?					.419
How often did you go to parties?					.507
How did you enjoy group fun?					.371

How difficult was it to speak in public?					.664
Eigen value	17.497	6.102	2.153	3.934	2.750
Cumulative predicted Percentage of the variance	21.461	28.231	42.679	33.254	38.248
Cronbach's alpha	0.709	0.734	0.785	0.778	0.752

Determining the reliability of the instrument for data collection: To determine the reliability of the study instrument, the internal consistency test was used and values of Cronbach’s alpha were calculated for 5 domains and are shown in Table 2.

## 4. Discussions

The face and content validity confirmed the simplicity and clarity of the questionnaire and evidences for the “relevance” of the statements obtained from the CVI showed a considerable degree of agreement (Mean=96.91) between opinions of the relevant specialists. Based on the results of this step, 156 items in 10 domains were confirmed. In this study, the correlation coefficient of the values obtained from the researcher-made instruments and the benchmark instruments was used for the criterion validation. The results showed the correlation between the two sets of instruments and confirmed that they measured the same subject (pregnant women’s lifestyle). However, the low correlation coefficient might be due to the fact that the researcher-made instruments included items other than most of the items in the benchmark instruments that could not be measured by the benchmark instruments. This caused the reduction of the correlation coefficient of the two sets of instruments and also showed the capacity of the researcher-made instruments for examining more items in the area of social determinants of health than the benchmark instruments.

The construct validity was assessed and confirmed through the exploratory factor analysis. The KMO index shows the appropriate correlation matrix for analyzing and confirms the factor analysis model. The cut-off point of 0.3 was considered as the minimum factor loading required for maintaining the statement. Then, the statement was allocated to one of the 5 factors that had the maximum factor loading. However, as explained before, 23 statements of different areas were deleted most of which related to nutrition. One reason may be the interval between initial and later samplings that resulted in different accessibility to certain foods or the fact that they were selected from different parts of the city. Cultural differences of the regions or people’s different levels of income led to not using certain foods or using some foods more than others. For instance, most of the foods with low nutritional value were placed in a class where they were not consistent with other factors in that class or they might have factor loading less than 0.3, so they were deleted. In their study on pschometry of healthy lifestyle scale, Taymoori et al. (2012) also referred to the effect of cultural differences on exclusion of certain questions about nutrition.

In this study, the Cronbach’s alpha coefficient of the questionnaire showed the high internal consistency of the statements and this confirmed the reliability of the questionnaire on pregnant women’s lifestyle with an approach to social determinants of health. Krageloh et al. (2011), Montazeri et al. (2003), and Seymour et al. (2001) also used internal consistency (Cronbach’s alpha) to determine reliability of their instruments.

Results of this study indicated that the designed instruments had the appropriate validity and reliability for examining pregnant women’s lifestyle with an approach to social determinants of health. Based on surveys, it seems that no instrument has been designed to measure the lifestyle with such an approach. The questionnaires used in other studies such as the life-style and personality inventory designed by Wheeler et al. in 1982 deal with Adlerian dimensions of lifestyle (Wheeler & Acheson, 1993). According to Adler, one develops his/her lifestyle in a way that is often useful in solving problems related to three duties of his/her life: social interactions with peers, occupation, and love (Lyons & Langille, 2000). Furthermore, Baldy’s lifestyle questionnaire is useful for evaluation of the lifestyle and lifetime overall health. The questionnaire has no false/true answers and measures 5 psychological dimensions of lifestyle including interpersonal, biological, social, cultural, and environmental (Behdani et al. 2000). In the revised version of the health- promoting life style profile, the focus is also on one’s inventive activities and cognition which act toward maintaining or enhancing the level of well-being, self-actualization, and personal satisfaction (Pakpour Hajiagha et al., 2012). Other questionnaires such as culture life- style inventory, children life-style scale-class, weight efficacy life style questionnaire, and lifestyle assessment have been either implemented using the translated versions of foreign instruments, or made by the researchers on the basis of the study objectives for limited needs and a specific time (Babaie & Saleh Sadeghpour, 2008). The only variables that are accounted for the majority of users’ behaviors are demographic-social variables such as the age, income, family size, and life cycle (Gust, 2004). The prevalent and rather common weakness of the available instruments is the failure to provide adequate information or inadequate psychometric analysis of instruments (Behdani et al., 2000; Mohammadi et al., 2005; Babaie & Saleh Sadeghpour, 2008). For instance, in Mohammadi et al’s study which was conducted to determine the validity and reliability of the health-promoting lifestyle inventory, it was concluded that those instruments required further psychometric analysis (Pakpour Hajiagha et al., 2012). In many studies (Yadollahi et al., 2006; Kamali Fard et al., 2010; Ashtiyani & kandovan, 2011) on lifestyle, it seemed that no appropriate psychometric analysis was performed on the instruments but only the content validity and the Cronbach’s alpha were determined without mentioning the details. On the contrary, the present study tried to provide sufficient information on validity, reliability, and designing of the instruments to inform the readers of the course of designing and assessment of the instruments. The positive points of these instruments include use of the target group’s opinions, diversity of specialists, simplicity and eloquence of the statements, and taking account of all areas related to the lifestyle. Normally, restricted subjects require less diversity of expertise. However, subjects like social determinants of health need more varieties of experts. Therefore, as explained before, once the appropriate statements were selected, specialists in different fields (statistics, social medicine, psychiatry, nursing, and social determinants of health) were requested to assess the scientific content of the statements. The diversity of fields resulted in the assessment of the designed instrument with different attitudes and opinions. However, one limitation of this study was the failure to compare those instruments with others due to lack of similar instruments with the same approach in Iran or in other countries or instruments which would be specific to pregnant women. The existing instruments on lifestyle only deal with evaluation of lifestyle’s behavioral manifestations, inventive activities and cognition of individuals, and visual studies of details of the lifestyle’s groups for a specific subject, or seek biological mechanisms of lifestyle. However, the present study questionnaire is looking for non-medical causes of lifestyle.

## 5. Conclusion

The pregnant women’s lifestyle measurement instrument was designed in the context of the Iranian culture and is suitable for studying the pregnant women’s lifestyle due to its appropriate validity, reliability, simplicity, and functionality in different situations.

## Footnotes

1 Pregnancy Risk Assessment Monitoring

2 Metabolic equivalent

3 Scale-level Content Validity Index/Averaging Calculation Method
